# Self-Consistent Mean Field Calculations of Polyelectrolyte-Surfactant Mixtures in Solution and upon Adsorption onto Negatively Charged Surfaces

**DOI:** 10.3390/polym12030624

**Published:** 2020-03-09

**Authors:** Eduardo Guzmán, Laura Fernández-Peña, Gustavo S. Luengo, Ana María Rubio, Antonio Rey, Fabien Léonforte

**Affiliations:** 1 Departamento de Química Física, Universidad Complutense de Madrid, 28040 Madrid, Spain; laura.fernandez.pena@ucm.es (L.F.-P.); anarubio@quim.ucm.es (A.M.R.); areygayo@ucm.es (A.R.); 2 Instituto Pluridisciplinar, Universidad Complutense de Madrid, 28040 Madrid, Spain; 3 L’Oréal Research and Innovation, 93600 Aulnay-Sous Bois, France; gustavo.luengo@rd.loreal.com

**Keywords:** polyelectrolyte, surfactants, mixtures, complexes, adsorption, calculations

## Abstract

Self-Consistent Mean-Field Calculations (SCF) have provided a semi-quantitative description of the physico-chemical behavior of six different polyelectrolyte-surfactant mixtures. The SCF calculations performed showed that both the formation of polymer-surfactant in bulk and the adsorption of the formed complexes onto negatively-charged surfaces are strongly affected by the specific nature of the considered systems, with the polymer-surfactant interactions playing a central role in the self-assembly of the complexes that, in turn, affects their adsorption onto interfaces and surfaces. This work evidences that SCF calculations are a valuable tool for deepening on the understanding of the complex physico-chemical behavior of polyelectrolyte-surfactant mixtures. However, it is worth noting that the framework obtained on the basis of an SCF approach considered an equilibrium situation which may, in some cases, be far from the real situation appearing in polyelectrolyte-surfactant systems.

## 1. Introduction

Mixtures formed by polyelectrolytes and surfactants have attracted the interest of many researchers during the last two decades [1,2,3,4,5,6,7,8,9]. This is due to the multiple applications of these systems, which range from the preparation of drug delivery systems to cosmetic products for hair care and conditioning, and from paints to different biotechnological products [10,11]. Despite the extensive development of studies involving polyelectrolyte-surfactant mixtures, there is an important lack of knowledge related to the relationships existing between the chemical nature and structure of the polyelectrolytes and surfactants and the complexation processes, as well as between the physico-chemical properties of such complexes and their adsorption at interfaces, with the understanding of the adsorption processes being essential in many of the applications of polyelectrolyte-surfactant mixtures [2,10,11]. This is because many of such applications, including the performance of hair care and conditioning formulations, mineral flotation, or drug delivery, rely on the interaction between polyelectrolyte-surfactant complexes and negatively-charged surfaces [2,12,13].

The most important drawback which restricts the progress of the understanding of the behavior of polyelectrolyte-surfactant mixtures is the strong controversy associated with the physico-chemical nature of the complexes formed in solution: equilibrium versus kinetically-arrested states [14,15,16,17,18,19,20,21,22,23]. It is worth mentioning that recent studies have suggested that the nature of the complexes may be related to the incorporation of counterions within the aggregates, with their absence favoring the formation of equilibrium complexes [24,25,26].

The general picture describing the complexation processes occurring in a mixture of a polyelectrolyte and a surfactant bearing opposite charges assumes that the addition of the charged surfactant to the oppositely-charged polyelectrolyte leads to the compensation of the polymer charges as a result of the binding of the surfactant molecules. This proceeds until the formation of polyelectrolyte-surfactant complexes, in which the charges of the polyelectrolyte chains are neutralized by the surfactant molecules; at this point, the poor colloidal stability of the neutral complexes drives the system to a phase separation region. It would be expected that the addition of surfactant amounts beyond the neutralization threshold would lead to a charge overcompensation, and hence to the re-dissolution of the complexes [8,27,28,29]. The above discussion relies on a complexation mechanism involving the existence of a true equilibrium upon mixing the polyelectrolyte and the surfactant [5]. However, there are some cases where the aggregation through a neutralization-overcompensation mechanism is not guaranteed, and the onset in the phase separation region occurs for compositions that are far from the neutralization threshold, with the polyelectrolyte-surfactant mixing protocol playing a key role in this type of phase separation [5,22,23,30,31,32,33,34,35,36,37]. This is associated with the formation of kinetically trapped aggregates, i.e., non-equilibrium complexes which evolve slowly towards the equilibrium state [5]. The formation of such kinetically trapped aggregates is the result of local surfactant concentration gradients (Marangoni stresses) that lead to the formation of compact aggregates, with a core which is supposed to be neutral, even though their net charge remains undercompensated [22,38,39]. Thus, such aggregates remain dispersed in the aqueous medium, with their sedimentation being slower than that corresponding to the true equilibrium complexes [18,19,38].

The formation of kinetically trapped aggregates may present a strong impact in the applications of these mixtures, enabling the shifting of the onset of the phase separation to compositions involving lower surfactant concentrations than those corresponding to the true equilibrium phase separation. This may be beneficial, from economic and eco-sustainability points of view, for applications involving the deposition of phase-separated aggregates, e.g., the performance of hair care and conditioning in cosmetics [2]. Despite the potential interest of kinetically trapped aggregates, their formation makes it difficult to introduce a thermodynamic description of the behavior of polyelectrolyte-surfactant mixtures, with theoretical calculations being a promising alternative for deepening on the thermodynamic bases, driving the aggregation and adsorption onto surfaces of polyelectrolyte-surfactant mixtures.

It is worth mentioning that given the strong controversy mentioned above about the origin of the complexation in a polyelectrolyte-surfactant mixture, theoretical calculations based on a Self-Consistent Mean-Field (SCF) approach can help give a better understanding of the physico-chemical framework describing the complexation process under conditions in which the thermodynamic equilibrium is ensured [40,41,42,43,44,45,46]. SCF calculations have allowed an understanding of the interactions occurring between polyelectrolytes and surfactants in aqueous medium, and the adsorption of the formed complexes onto negatively-charged solid surfaces [47]. In particular, SCF calculations for pseudo-binary mixtures formed by poly(diallyl-dimethylammonium chloride) (PDADMAC) and up to five different surfactants, with such surfactants presenting different chemical nature (anionic, zwitterionic, and non-ionic) have been performed. The specific choice of the mixtures of PDADMAC and different surfactants is related to the interest of these systems in the cosmetic industry, and in particular in the fabrication of hair care and conditioning products. Furthermore, the adsorption of such mixtures onto negatively-charged surfaces is important because hair, upon bleaching or weathering, presents a negative surface charge, which is the substrate of cosmetic product. This makes it necessary to analyze the adsorption of polyelectrolyte-surfactant mixtures onto model surfaces, which might help with the comprehension of the physico-chemical bases underlying the performance of cosmetic formulations. Therefore, the incorporation of a molecularly detailed model for both the surfactants and the polymers may provide important insights on the physico-chemical bases of the performance of cosmetic formulations. For this purpose, the Scheutjens–Fleer SCF theory, which provides a coarse-grained description of polymers and surfactants presenting complex architecture without loss of molecular and relevant realistic details, has been used [46,48]. It may be expected that these SCF calculations may be used as a semi-quantitative prediction tool for unravelling the most probable structures of pseudo-binary polyelectrolyte-surfactant systems in the bulk and upon adsorption onto a solid substrates [5,45]. This work shows that the SCF is a versatile tool for the prediction of the behavior of a wide range of polyelectrolyte-surfactant mixtures, regardless of the structure and properties of the surfactant.

## 2. Methods 

The SCF theory is a powerful tool enabling the description of the self-assembly of complex colloidal structures [49,50,51,52,53] based on the minimization of a mean-field free-energy functional, which requires the introduction of three parameters (see Table 1 for values) as input of the model: (i) the short-range interaction parameters (Flory–Huggins parameters *χ*) between segments of molecules, (ii) the relative dielectric permittivity (*ε*), and (iii) the valence (*ν*). The dielectric permittivity and the valence account for the electrostatic contributions, whereas the Flory–Huggins parameters are key for the formation and stability of the self-assembly colloids. The values of the Flory–Huggins parameters were adapted from Postmus et al. [54], and those corresponding to the relative dielectric permittivities were chosen to ensure a proper representation of the heterogeneities in the hydrophobicity existing within the polyelectrolyte-surfactant complexes. The calculations performed took into account the molecular detail, the pH, and the ionic strength. In the following, the most fundamental aspects related to the use of SCF theory for the theoretical prediction of the self-assembly in solution of polyelectrolyte-surfactant complexes and their adsorption onto negatively charged surfaces will be included. Further details about the use of the SCF theory can be found elsewhere [43,46,48,54,55].

The use of SCF calculations relied on the discretization of the molecules, following the scheme proposed by Scheutjens and Fleer [43,46,48]. This was done in terms of, lattice size, i.e., the size of the atoms which composed the molecules, which was fixed at *a* = 0.3 nm [56,57]. The central idea of SCF calculations is to replace the interactions occurring within an ensemble of molecules by the interaction of an effective molecule within a field, thus approximating a many-body problem as a one-body problem. The mean-field free-energy functional can be written in terms of the molecule’s segment density profiles for a specific segment type, *φ_X_*(*z*), and the conjugate segment potential profiles, *u_X_*(*z*), with *X* referring to a segment type and *z* indicating the spatial coordinate. The model introduced the water hydrogen-bonding capability, mainly with oxygen and hydroxyl groups of sugar rings, using a five-site description of water molecules, and negative Flory–Huggins parameters that ensured the solubility of the considered species. For the sulfonate groups, a five-sites representation was also used, as well as for cationic groups of PDADMAC and zwitterionic surfactant, i.e., N. The inclusion of a detailed molecular structure, and especially those aspects related to the hydrogen bonding of the complexes, played an important role for a proper description of the phase behavior of polyelectrolyte-surfactant complexes, as was recently stated by Ali et al. [58]. The optimization process was based on a numerical SCF procedure which allowed us to relate the volume fractions and the potentials following the basic principles described in the literature [43,51,56,57]. It is worth mentioning that the volume fraction presents a key role in the development of the SCF calculation because it allows one to derive other parameters, including the electrostatic potential, the charge distribution, and the local dielectric permittivities [51].

The molecular partition functions for single chains play a central role in the mean field free-energy. From an operational point of view, it was convenient to use the dimensionless form of the Edward diffusion equation [59]:

(1) $$\frac{\partial G\left(z,s\right)}{\partial s}=\frac{1}{6}\frac{\partial^{2}G\left(z,s\right)}{\partial z^{2}}-u\left(z,s\right)G\left(z,s\right),$$

where *s* and *u*(*z,s*) refer to the number of segments and the volume fraction for a given segment potential, respectively. *G*(*z,s*) represents the end-point distribution for a chain segment, which provides information about the probability that the end point of a walk formed by *s* segments ends up at position *z*. *G*(*z,s*) can be assumed to be the Boltzmann weight of the field [41]. Equation (1) is solved using as boundary conditions: (i) a hydrophilic surface exists at *z* = 0, and (ii) the behavior of the mixture is similar to that found in the bulk at large *z* values. The SCF approach requires the use of freely-jointed chains (FJC) to map the Edward’s equation over a lattice, i.e., considers freely rotating bonds with the same length [60]. The use of this FJC model is preferred because it allows one to obtain the partition function and the volume fraction for a given segment potential *u*(*z,s*) [41].

The segment potentials can be computed when the volume fractions are available. The Flory–Huggins-like interaction parameter and a contribution to ensure the compressibility of the system were used to calculate the interaction energy of a segment at a specified location. The estimation of the number of segment-segment contacts was calculated using the Bragg–Williams mean-field approximation [61]. Furthermore, terms accounting for the long-range electrostatic interactions must be included in the segment potential, which makes it necessary to solve the Poisson equation:

(2) $$\frac{\partial \varepsilon \left(z\right)}{\partial z}\frac{\partial \psi \left(z\right)}{\partial z}=-q\left(z\right),$$

where *ε*(*z*) and $\frac{\partial \psi \left(z\right)}{\partial z}$ represent the dependences of the dielectric constant on the position and the electrostatic potential *Ψ*(*z*) gradient, respectively. *q*(*z*) defines the charge density *q*(*z*) = ∑*φ_X_*(*z*)*ev_X_*, where *e* denotes the elementary charge and *v_X_* the valence, including the sign for the segment *X* (see Table 1). As aforementioned, at *z* = 0 a hydrophilic negatively-charged solid surface is placed as substrate for the adsorption from the bulk. The analysis of the adsorption onto the surface required initially performing the optimization of the bulk composition and aggregation following the methodology described by Banerjee et al. [57]. Thus, the calculation procedure can be summarized in the following two steps: (i) building of binding isotherms, considering the binding of polyelectrolyte chains to spherical micelles, and (ii) equilibration of the binary polyelectrolyte-surfactant mixture at fixed chemical potentials with a negatively-charged silica surface (~200 mC/m^2^, the surface is referred to as *Si* in Table 1). For the spherical micelles, the grand potential of the composite system was kept fixed at a value higher than that associated with the formation of the first micelle, i.e., all the calculations were performed for surfactant concentrations above the critical micelle concentration (cmc). All the calculations were performed for charge ratio *f* < 0.5, fixing the PDADMAC concentration at 0.2 wt % and increasing the surfactant concentration in the micelles. The latter involved polymer concentration in the complexes also increasing. It should be noted that the value of the grand potential per micelle does not affect significantly the extracted conclusions; higher values would give rise to a lower micelle concentration, whereas a lower one would be associated with a higher micelle concentration. It is worth mentioning that the change in the micelle concentration had little effect on the surfactant chemical potential, on the micelle size and stability, or on the capacity for PDADMAC-surfactant binding. From the calculations, the volume fraction profiles for all the segments of type *i* at coordinate *z*, $\phi_{i}\left(z\right)\;\;$ may be obtained, with the adsorption being quantified in terms of the excess adsorption $\theta_{i}^{\sigma }=\sum_{z=1}^{M}\left[\phi_{i}\left(z\right)-\phi_{i}^{b}\right]\;$, where $\phi_{i}^{b}$ is the volume fraction in the bulk obtained from SCF optimization calculations [56].

**Figure 1:**
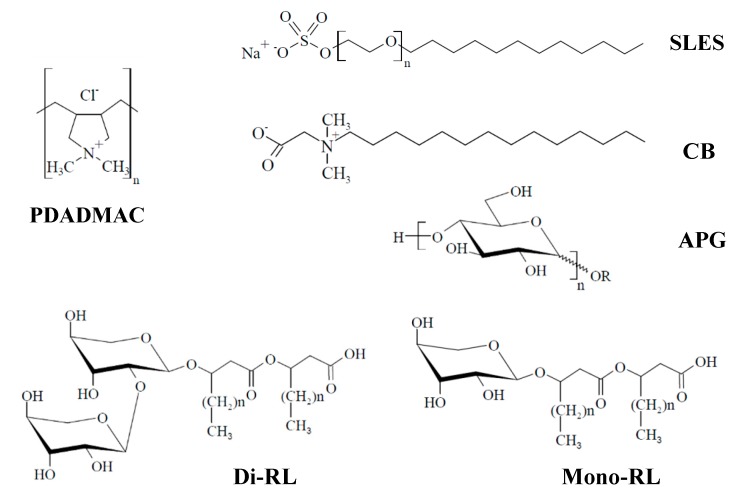
Molecular structures for the polyelectrolyte and the surfactants used in this study, *n* for SLES assumes a value of 5 and for APG, di-RL, and mono-RL a value of 10.

The procedure described above was used for describing the behavior of mixtures formed by PDADMAC containing 100 monomers, and different surfactants: (i) three anionic, namely sodium laureth sulfate (SLES), di-rhamnolipid (di-RL), and mono-rhamnolipid (mono-RL), (ii) the zwitterionic cocobetaine (CB), and (iii) the neutral alkyl polyglucoside (APG), under conditions in which the ionic strength was fixed by the addition of KCl and the pH at 5.6. This work paid interest to the study of the polyelectrolyte-surfactant mixtures where the ionic strength was fixed using KCl. However, it should be expected that the nature of the counterions may present a decisive impact in the interactions between the polymer and the surfactant molecules [62]. This may lead to changes in the complexation process, which allow one to tune the nature of the aggregates formed upon mixing the polyelectrolyte and the surfactant, as was stated by different works [15,18,19,20,21,24,25,30]. Figure 1 summarizes the chemical structures of the polyelectrolyte and the studied surfactants.

## 3. Results and Discussion 

### 3.1. SCF Calculations of Pseudo-Binary Polyelectrolyte-Surfactant Mixtures in the Bulk

**Figure 2:**
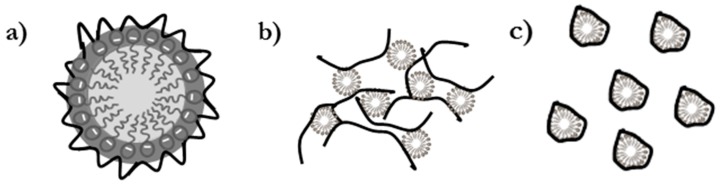
(**a**) Scheme representing the model assumed for Self-Consistent Mean-Field Calculations (SCF) calculations. (**b**) Scenario for those systems in which micelles bridge several polymer chains (formation of multichain complexes, *g_PS_* > 1). (**c**) Scenario for those systems in which micelles do not bridge polymer chains (*g_PS_* < 1).

SCF calculations can help give a better understanding of the self-assembly of pseudo-binary polyelectrolyte-surfactant mixtures in solution. The model assumed that the formation of polyelectrolyte-surfactant (P-S) complexes occurs as result of the binding of polymer chains to preformed surfactant micelles, i.e., the formed complexes may be considered as surfactant micelles decorated with PDADMAC chains. Therefore, it was expected that the hydrophobic groups of the surfactants would remain in the inner region (core) of the aggregates, whereas the polar hydrophilic heads of the surfactants would form a corona in whose edges were placed the PDADMAC chains [63] (see Figure 2a). 

**Figure 3:**
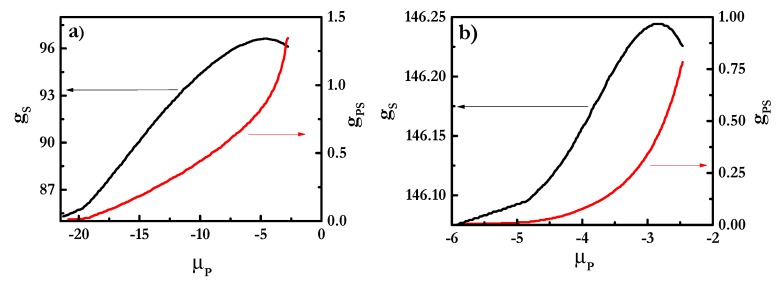
Dependences of the aggregation number of surfactants in the complexes (*g_s_*, left axis) and the degree of polyelectrolyte–surfactant binding (*g_PS_*, right axis) on *μ_P_* for mixtures of PDADMAC with SLES (**a**) and with CB (**b**).

Figure 3 shows the binding isotherms for mixtures of PDADMAC with SLES (Figure 3a) and CB (Figure 3b). These isotherms considered the formation of polyelectrolyte-surfactant complexes where spherical micelles of the surfactant formed a core surrounded by the polyelectrolyte chains, as is sketched in Figure 2a. It is worth mentioning that other geometries for the micelles, which also lead to stable complexes (cylindrical for all the mixtures and lamellar structures when mixtures including rhamnolipids are concerned), were considered. However, for the sake of simplicity, this work is focused on mixtures where the surfactant concentration is not high enough to distort the spherical geometry of the micelles and the discussion will be focused on this particular geometry. On the basis of the binding isotherms, it is possible to evaluate the assembly process of polyelectrolyte-surfactant complexes in terms of the evolution of the aggregation number of the surfactant in the complexes, *g_S_*, and the degree of polyelectrolyte-surfactant binding, *g_PS_*, i.e., the ratio between the number of monomers and surfactant charges, with the increase in the chemical potential of the polymer, *μ_P_*, i.e., the polymer concentration [56].

**Figure 4:**
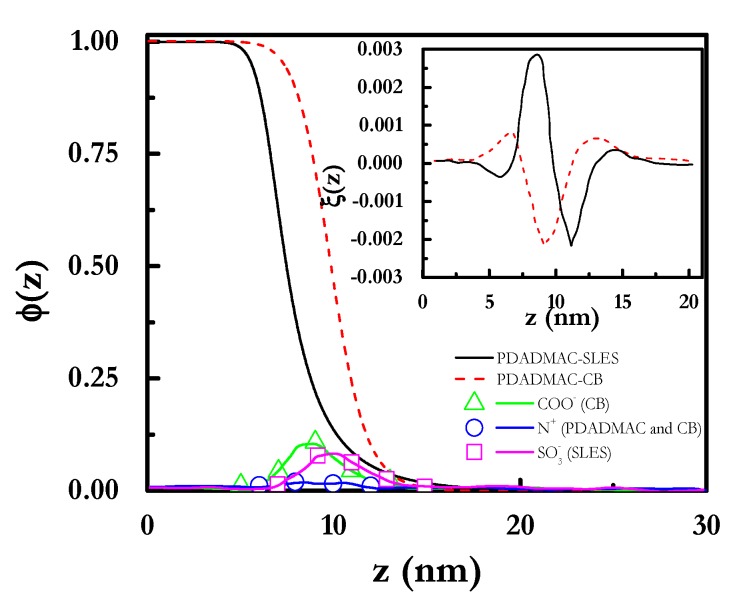
Radial volume fraction (*φ*(*z*)) profiles for the inner hydrophobic core and the charged corona of polyelectrolyte–surfactant complexes. The polyelectrolyte had a length of 100 segments and a segment valence *ν_p_* = 1. The inset represents the dimensionless radial charge density (*ξ*) profiles for the complexes.

The results show differences in the aggregation behavior depending on the surfactant nature, which may be the result of the differences existing in the formation of polyelectrolyte-surfactant complexes, depending on the nature of the surfactants [39]. The aggregation number of surfactants in the complexes increased with the chemical potential of the polymer *μ*_P_ for mixtures of PDADMAC with both surfactants until a threshold value of *μ*_P_, which depended on the surfactant nature. The initial increase was a signature of the cooperative binding of the polymer chains to the surfactant micelles, i.e., an increase in the polymer concentration increases progressively with the number of surfactant molecules in the complexes [57]. This may be rationalized by considering that as the polymer concentration in the complexes increases the number of molecules needed for obtaining neutral complexes also does. Paying attention to the dependence of the surfactant aggregation number for PDADMAC-SLES mixtures, it was found that close to the charge neutralization, i.e., for a stoichiometric composition of the mixture, *g_S_* started to decrease with the increase in the chemical potential of the polymer. This is explained by considering that the spherical shape of the surfactant micelles starts to be compromised and, as a consequence, the colloidal stability may be lost when the polyelectrolyte-surfactant binding overcomes the threshold value of 1 (see Figure 3b). This is the result of the adsorption of the PDADMAC chains at the outer shell of the micelles, as evidenced by the radial volume fraction profiles, *φ* (see Figure 4), with the hydrophobic core presenting a homogeneous density of about 1, and the charged groups, including those corresponding to PDADMAC, remaining located at the periphery. The polymer binding did not affect significantly the profile of the micelle, and only in PDADMAC-SLES mixtures could a slight penetration of some segments of the polymer to the inner region of the micelles be expected. Thus, the existence of loops and tails on the PDADMAC chains in the outer shell of the micelle enabled the bridging between neighboring micelles, which prevented colloidal stability. Therefore, the increase of the polymer-surfactant binding up to values well above 1 was compatible with the formation of PDADMAC-SLES complexes in which some polymer chains could be shared between more than one micelle, and micelles can act as bridges between several polymer chains (see Figure 2b), in agreement with the experimental findings by Hoffman et al. [64,65] for the assembly of oppositely-charged polyelectrolytes and surfactants. The above described bridging phenomenon suggests that each polyelectrolyte chain does not compensate the charges corresponding to a single SLES micelle, and different segments of the PDADMAC chains may protrude from the outermost region of the micelle, where they are bound, to the solution. This enables the binding of a polymer chain to several micelles, which may be considered analogous to the formation of a pearl-necklace-like structure. 

The scenario found for the PDADMAC-CB mixtures (see Figure 3b) was qualitatively analogous to that occurring in mixtures containing SLES, even though some subtle modifications associated with the different characteristics of the surfactant were found. For mixtures containing CB, the degree of binding remained well below 1, even for the highest values of *μ_P_*, which may be considered a signature of an electrostatically-hindered binding, probably associated with the zwitterionic character of CB [66]. This may be considered a signature of the formation of complexes where each polymer chain is only bound to one micelle (see Figure 2c). The influence of the electrostatic repulsion was also clear from the increase of the binding at higher values of the polymer concentration than in PDADMAC-SLES mixtures. Despite the absence of real charge compensation in PDADMAC-CB mixtures, a reduced colloidal stability was also found with the increase of *μ*_P_.

The analysis of the radial volume fraction (*φ*(*z*)) profiles (see Figure 4) obtained for the two pseudo-binary mixtures showed that despite the polyelectrolyte chains adsorbed at the outermost region of the micelles (average diameter around 7 nm and 9 nm for mixtures containing SLES and CB, respectively), some polymer segments, mainly in PDADMAC-SLES mixtures, could penetrate inside the micelle, resulting in the formation of complexes with a fuzzy structure. This justifies the binding between the polyelectrolyte and the surfactant in such a way that several polyelectrolyte chains can be attached to one micelle when the polymer concentration increases (*g_PS_* > 1). The dimensionless charge density (*ξ*(*z*) = *q*(*z*)/*e*) profiles (inset in Figure 4) show the absence of real neutralization of the polyelectrolyte charges due to the binding of CB, whereas a stronger interaction was found for PDADMAC–SLES mixtures. This agrees with the experimental scenario found for pseudo-binary PDADMAC-SLES and PDADMAC-CB mixtures, with the former one showing a clear binding of the anionic surfactant to the charged monomer of the PDADMAC from the lowest values of the surfactant concentration [66,67], whereas for PDADMAC-CB mixtures the electrostatic hindered the association process up to surfactant concentrations close to the critical micelle concentration of the CB [66]. 

**Figure 5:**
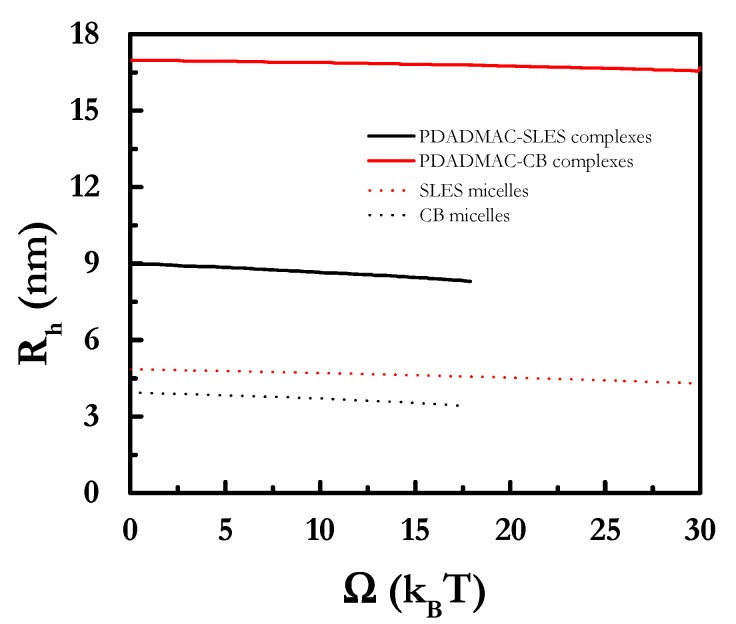
Grand potential *Ω* dependences of the hydrodynamic radius, *R_h_*, for the polymer–surfactant complexes and the surfactant micelles in absence of polyelectrolyte.

SCF calculations also gave access to the size of the self-assembled structures. Figure 5 reports the hydrodynamic radius, *R_h_*, corresponding to polyelectrolyte–surfactant complexes and to surfactant micelles in the absence of polyelectrolyte as a function of the free energy associated with the formation of micelles (grand potential *Ω*). *R_h_* was not significantly affected as a result of the increase in the grand potential. This may be explained as a consequence of the formation of spherical aggregates with a size defined by the maximum effective packing (optimal size) to ensure their stability. 

The results show that CB micelles are bigger than those of SLES. Therefore, a more important role of the electrostatic repulsion between the heads may be expected for the zwitterionic of CB than for SLES, which leads to the increase in the micellar size. This should play a main influence in the self-assembly process of polyelectrolyte–surfactant mixtures. It is worth mentioning the absence of qualitative agreement between the values of the hydrodynamic radius obtained on the basis of the SCF calculations and those obtained experimentally using dynamic light scattering [39,66,67]. This apparent discrepancy may be explained by considering that the hydrodynamic radius obtained by SCF calculations was actually the measurement of the minimum distance in which it is likely to find two micelles decorated with polymers. This distance was lower for PDADMAC–SLES than for PDADMAC–CB mixtures, which may have resulted from the higher trend of the former system to self-assembly, leading to the formation of aggregates involving several polyelectrolyte chains. The above results provide evidence of the importance of the surfactant nature, and the interactions involved in the assembly of polymer-surfactant complexes, as well as the semi-quantitative agreement between the SCF calculations and the previous studies on the bulk aggregation of similar systems [39,66,67,68]. Deepening on this aspect, the binding isotherms obtained for other polyelectrolyte-surfactant mixtures will be discussed in the following. 

**Figure 6:**
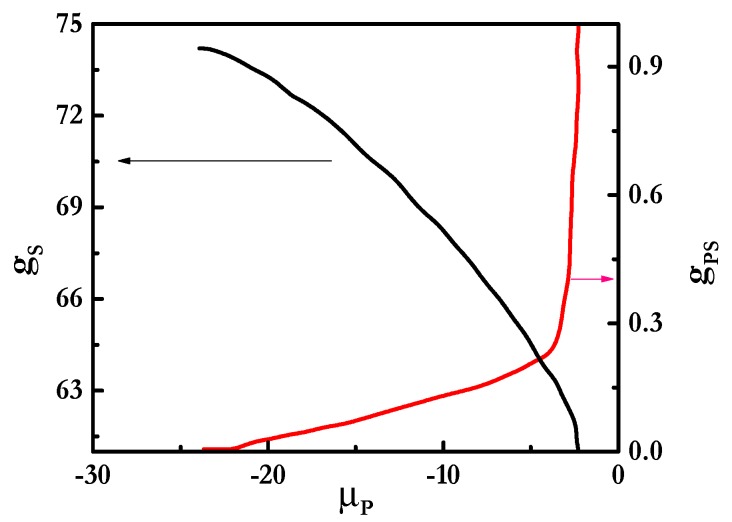
Dependences of the aggregation number of surfactants in the complexes (*g_s_*, left axis) and the degree of polyelectrolyte–surfactant binding (*g_PS_*, right axis) on *μ_P_* for mixtures of PDADMAC with APG.

Figure 6 shows the isotherms obtained for mixtures of PDADMAC and a neutral surfactant APG. On the contrary to that found either with SLES or CB, no electrostatic interactions between PDADMAC and APG can be expected, which make it necessary to consider the role of the interactions between the hydrophobic tails of APG and the hydrophobic domains within the PDADMAC backbone. As a result, a clear non-cooperative association between the polyelectrolyte and the surfactant was found for PDADMAC-APG mixtures, as evidenced by the strong decrease of *g_S_* with the chemical potential of the polymer. The difference in the cooperativity character of the binding between mixtures of PDADMAC with the anionic SLES and the zwitterionic CB and those of PDADMAC with APG may be related to the different nature of the interactions involved in the process. Thus, it would be expected that electrostatic interactions may enhance the cooperativity of the binding in relation to that which happens for those cases where non-electrostatic binding is the dominating factor. The change in the nature of the interactions driving the self-assembly of the polyelectrolyte-surfactant aggregates may lead to changes in the structure, as is expected from the different binding isotherms [69]. 

**Figure 7:**
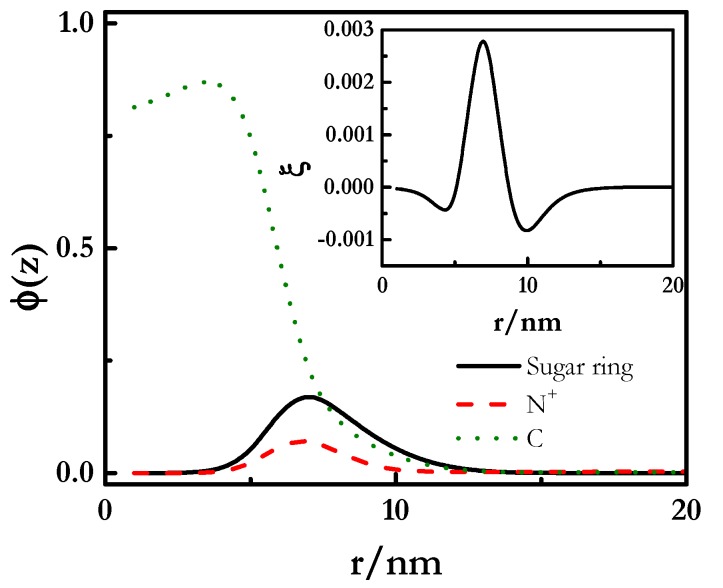
Radial volume fraction (*φ*(*z*)) profiles for the inner hydrophobic core and the charged corona of PDADMAC-APG complexes. The polyelectrolyte had a length of 100 segments and a segment valence *ν_p_* = 1. The inset represents the dimensionless radial charge density (*ξ*) profiles for the complexes.

The lack of electrostatic interactions is clearly evidenced from the absence of electroneutralization in the complexes, i.e., *g_PS_* did not reach the value of 1, even though there was a continuous increase in the binding with the chemical potential of the polymer. A deeper understanding of this may be obtained from the analysis of the radial volume fraction profile shown in Figure 7. This shows that the polyelectrolyte always remained at the outer shell of the spherical micelles, even though the mostly positive values found for the dimensionless charge density profiles (inset in Figure 7) are a clear confirmation of the absence of real charge compensation, as is expected from the neutral character of APG, in agreement with previous experimental results [38]. Thus, the presence of some uncompensated charges makes the formation of stable complexes possible over a longer composition range than those involving a charged surfactant such as SLES, yielding a behavior similar to PDADMAC-CB mixtures. Even though the stability of complexes is almost guaranteed in a wider range of compositions, the increase in polymer in the complexes can lead to a worsening of the stability, as a result of the depletion of the surfactant from the micelles, or by a bridging phenomenon through the formation of hydrogen-bonds between the head groups of APG. 

A detailed analysis of the radial volume fraction profiles (see Figure 7) suggests that PDADMAC-APG complexes present the sugar rings at the periphery of the aggregates, and the association occurs through hydrophobic interactions between the alkyl tail of the surfactant molecules and the methyl groups surrounding the ammonium group of PDADMAC. This may enable the formation of complexes where different polymer chains are incorporated as a result of the hydrogen bonds formed between the hydroxyl groups of different surfactant molecules bound to some polymer chain. The scenario found for mixtures containing APG is slightly modified when this surfactant is replaced by a surfactant in which the sugar hydrophilic head presents a charged group as occurs when di-RL is concerned. It would be expected an aggregation with reminiscences of the behavior of a surfactant containing a negatively charged surfactant, such as SLES, and APG. 

**Figure 8:**
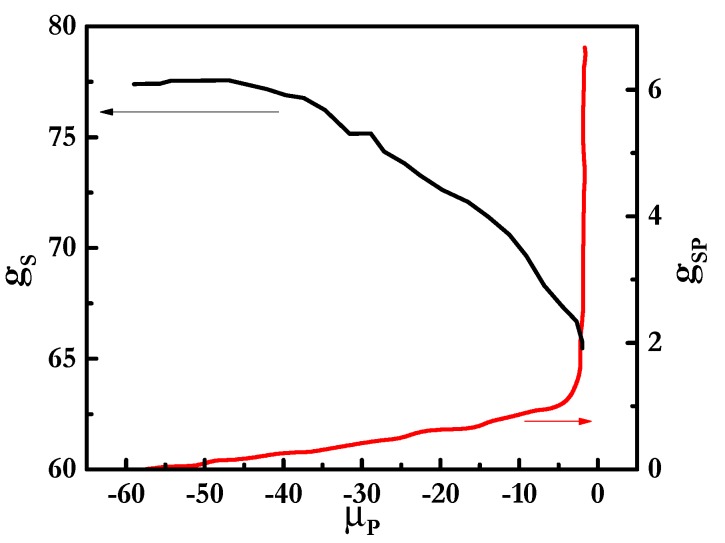
Dependences of the aggregation number of surfactants in the complexes (*g_s_*, left axis) and the degree of polyelectrolyte–surfactant binding (*g_PS_*, right axis) on *μ_P_* for mixtures of PDADMAC with di-RL.

Figure 8 shows the binding isotherm for mixtures involving the di-RL. The results show clearly that even though the surfactant contained a charged group, a clear non-cooperative binding was also found, which makes the association process different to that found for mixtures of PDADMAC with SLES or CB. However, the strong increase in *g_PS_* with the increase in *μ_P_* is a clear signature of the existence of micelles bridging several polymer chains when the di-RL is combined with PDADMAC. From the results discussed above, it is clear that di-RL combines in its association some features of the charged surfactants with others typical of neutral ones.

**Figure 9:**
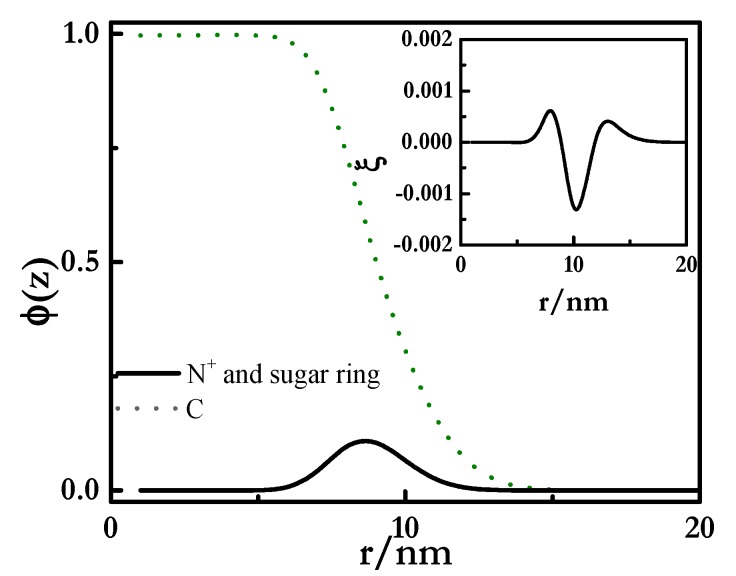
Radial volume fraction, *φ*(*z*), profiles for the inner hydrophobic core and the charged corona of PDADMAC-di-RL complexes. The polyelectrolyte had a length of 100 segments and a segment valence *ν_p_* = 1. The inset represents the dimensionless radial charge density (*ξ*) profiles for the complexes.

**Figure 10:**
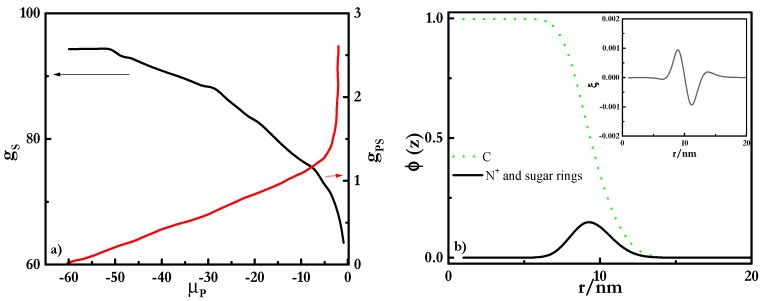
(**a**) Dependences of the aggregation number of surfactants in the complexes (*g_s_*, left axis) and the degree of polyelectrolyte–surfactant binding (*g_PS_*, right axis) on the chemical potential of the polymer *μ_P_* for mixtures of PDADMAC with mono-RL. (**b**) Radial volume fraction (*φ*(*z*)) profiles for the inner hydrophobic core and the charged corona of PDADMAC-mono-RL complexes. The polyelectrolyte had a length of 100 segments and a segment valence *ν_p_* = 1. The inset represents the dimensionless radial charge density (*ξ*) profiles for the complexes.

The analysis of the radial volume fraction profiles and the density of the dimensionless charge density shown in Figure 9 evidences that the structure of the formed complexes for the di-RL was similar to that found for PDADMAC-SLES mixtures, even though some participation of the hydrogen bonds between the sugar rings may also contribute to the stabilization of the complexes. The increase in the hydrophobicity of the rhamnolipid by changing the two rhamnose rings of the hydrophilic head with a single one (mono-RL, see results in Figure 10) shows that the increase in the hydrophobicity only shifts slightly up to higher values of the concentration of surfactant needed for obtaining the complexes involving several polymer chains, which agrees with the experimental framework discussed in our previous publication [38].

### 3.2. SCF Calculations of Polyelectrolyte-Surfactant Mixture Adsorption onto a Solid Interface

**Figure 11:**
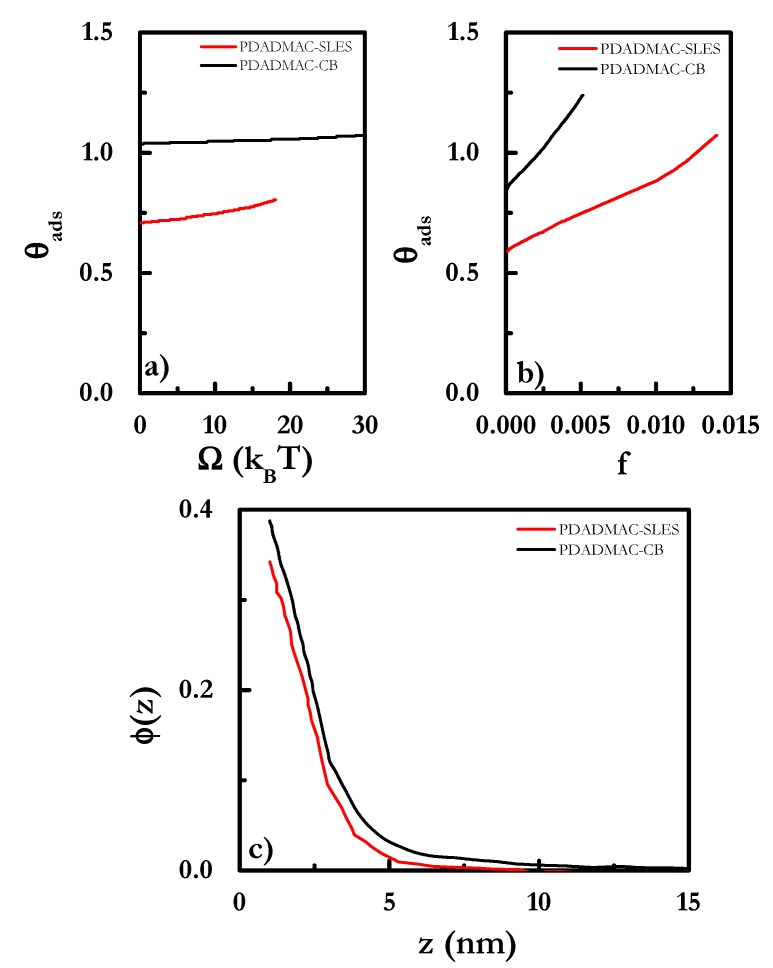
(**a**) Grand potential (*Ω*) dependence of the coverage, *θ_ads_*, for different polyelectrolyte–surfactant mixtures. (**b**) *f*-ratio, which defines the ratio between the number of monomers and the number of surfactant molecules, dependence of the coverage, *θ_ads_*, dependences for different polyelectrolyte–surfactant mixtures. (**c**) Volume fraction profiles, *φ*(*z*), as a function of the distance *z* from the surface for the different studied mixtures.

SCF calculations were used in the study of the adsorption of two of the mixtures, those of PDADMAC with CB and SLES, onto negatively-charged solid surfaces. Figure 11 shows the adsorption isotherms corresponding to the polymer–surfactant mixtures.

The dependences in the coverage on the grand potential *Ω* and the *f*-ratio (the ratio between the number of monomers and the number of surfactant molecules) confirmed the trend found for the total adsorbed amount, as was observed using ellipsometry in our previous study [66], i.e., the adsorbed amount with the surfactant concentrations. However, SCF calculations predicted higher adsorptions for PDADMAC-CB mixtures than for PDADMAC-SLES ones, which differs from the experimental findings. This may result from the fact that the calculated adsorption isotherms were based on the equilibration of polyelectrolyte-surfactant aggregates where surfactant micelles were decorated by surfactant, and hence the deposition occurred through the deposition of such structures directly onto the surface. This led to a situation where the electrostatic interactions between the surface and the polymer at the outermost region of the micelles was the driving force for the deposition, with this deposition mechanism resulting in a higher deposition as the size of the complexes increased. Similar conclusions may be extracted from the volume fraction profiles shown in Figure 11c, where it is clear that layers containing CB were extended to larger distances from the surface than those formed by PDADMAC-SLES mixtures.

## 4. Conclusions

This work analyzed, using SCF calculations, the self-assembly in solution of polyelectrolyte-surfactant mixtures, and the adsorption of the formed complexes onto negatively-charged surfaces. The obtained predictions correspond to systems in which the minimum Gibbs free energy has been achieved, with this being a situation which in many cases was far from the experimental scenario, as a result of the frequently found non-equilibrium character of the assembly of polyelectrolyte-surfactant systems. However, this study showed that even though the real situation may not be a true equilibrium, SCF calculations give a semi-quantitative prediction of the framework found in experimental studies dealing with the physico-chemical behavior of pseudo-binary polyelectrolyte-surfactant mixtures, both in the bulk and upon adsorption at the solid-liquid interface. SCF calculations showed that both electrostatic and non-electrostatic interactions may influence the assembly of polyelectrolyte-surfactant complexes in solution, which facilitates the formation of complexes between PDADMAC and anionic, zwitterionic, and non-ionic surfactants. However, the nature of the surfactant impacts decisively on both the complexation mechanism and the physico-chemical properties of the obtained complexes, with this latter governing the deposition of the complexes onto negatively-charged surfaces. Furthermore, the formation of complexes where surfactant micelles act as bridges between different PDADMAC chains was expected, according to results obtained from the performed calculations. More detailed models are required for obtaining a quantitative comparison between experiments and calculations. However, SCF allowed predictions to be made which can be useful for the development of in silico approaches for designing new products based on polyelectrolyte-surfactant mixtures.

## Figures and Tables

**Table 1:** Flory–Huggins interaction parameters, χ, between various pairs of segments, relative dielectric constant, ε, and valence, ν, of the segment types, as used in the SCF calculations. Note that the segment type X denotes the deprotonated segment of a carboxylic group, and Si and *w* denote the surface and the water, respectively.

*χ*	*w*	*C*	*O*	*S*	*N*	*K*	*Cl*	*OH*	*X*	*Si*	*ε*	*ν*
*w*	0	1.6	−0.6	0	0.5	0	0	−0.6	0	1	80	0
*C*	1.6	0	1.6	2	2	2	2	2	2	0	2	0
*O*	−0.6	1.6	0	0	0	0	0	0	0	0	1.5	0
*S*	0	2	0	0	0	0	0	0	0	0	3.4	−0.2
*N*	0.5	2	0	0	0	0	0	0	0	0	7	0.2
*K*	0	2	0	0	0	0	0	0	0	0	6.1	1
*Cl*	0	2	0	0	0	0	0	0	0	0	6.1	−1
*OH*	−0.6	2	0	0	0	0	0	0	0	0	1.5	0
*X*	0	2	0	0	0	0	0	0	0	0	1.5	−0.2
*Si*	1	0	0	0	0	0	0	0	0	0	5	−0.1
